# Supplementary material for: Updated Phylogeny of Chikungunya Virus Suggests Lineage-Specific RNA Architecture
Source: Viruses. 2019 Aug 29;11(9):798. doi: 10.3390/v11090798 (PMC6784101; doi:10.3390/v11090798)
Supplement: Supplementary file 1 [file viruses-11-00798-s001.pdf]

Updated phylogeny of Chikungunya virus suggests lineage-specific RNA architecture – Supplement –

Adriano de Bernardi Schneider[1,†], **Roman Ochsenreiter**[2,†], **Reilly Hostager**[1], **Ivo L. Hofacker**[2,3], **Daniel Janies**[4], **and Michael T. Wolfinger**[2,3,✉]

[1] AntiViral Research Center, Department of Medicine, University of California San Diego, San Diego, 92103, United States of America
[2] Department of Theoretical Chemistry, University of Vienna, Währingerstraße 17, 1090 Vienna, Austria
[3] Research Group BCB, Faculty of Computer Science, University of Vienna, Währingerstraße 29, 1090 Vienna, Austria
[4] Department of Bioinformatics and Genomics, University of North Carolina at Charlotte, Charlotte, 28223, United States of America
[†] These authors have contributed equally to this work.

Correspondence: ***michael.wolfinger@univie.ac.at***

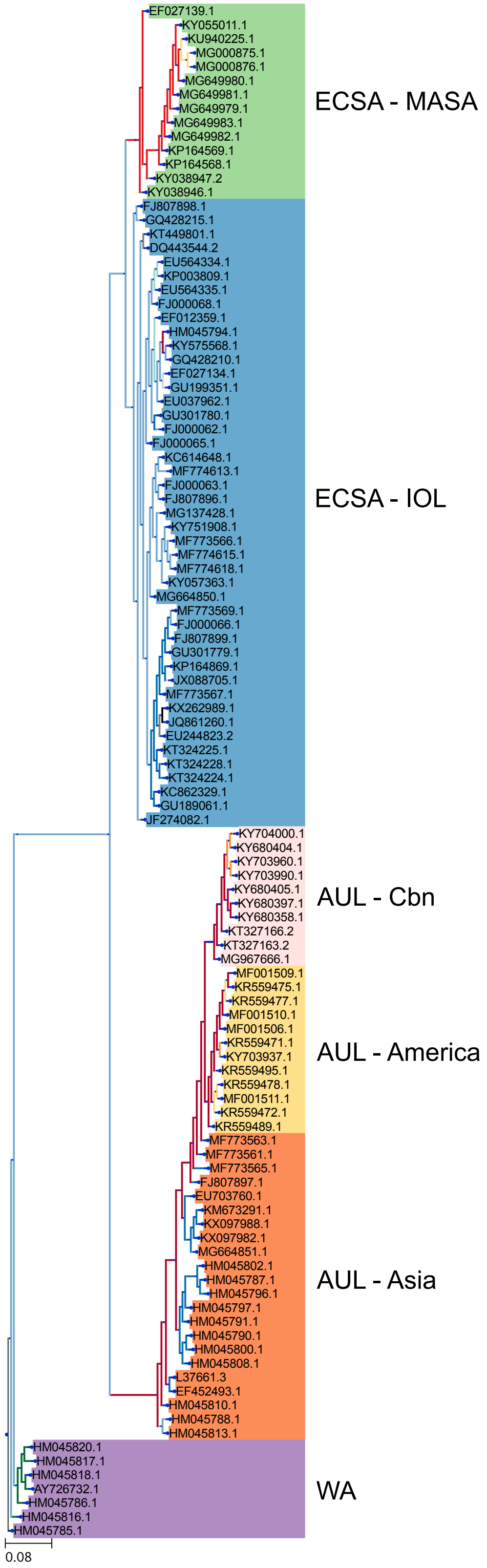

Fig. 1. Maximum-Likelihood phylogenetic tree based on a manually curated alignment of 111 complete CHIKV 3'UTRs. Color coding of the tree based on Fig. 1 in main text. Leaves are colored according to lineage, with UTRs harbouring the duplication event in the AUL-American lineage are colored separately.
